# IUC24434-82 Real-world use of abiraterone and olaparib in metastatic castration-resistant prostate cancer (mCRPC): a 2-center UK experience

**DOI:** 10.1093/oncolo/oyaf248.038

**Published:** 2025-08-29

**Authors:** M Ali Hassan, J Kadamala Samuel, M Maffezzoli, N Kolambil Saidalavi, R Arora, S Sandulache, F Di Costanzo, A Maniam, G Luigi Banna

**Affiliations:** Portsmouth Hospital University NHS Trust, United Kingdom; Portsmouth Hospital University NHS Trust, United Kingdom; Portsmouth Hospital University NHS Trust, United Kingdom; Portsmouth Hospital University NHS Trust, United Kingdom; Portsmouth Hospital University NHS Trust, United Kingdom; Portsmouth Hospital University NHS Trust, United Kingdom; Newcastle Hospitals NHS Foundation Trust, United Kingdom; Portsmouth Hospital University NHS Trust, United Kingdom; Portsmouth Hospital University NHS Trust, United Kingdom

## Abstract

**Background:**

Metastatic castration-resistant prostate cancer (mCRPC) remains a therapeutic challenge, with limited long-term treatment options. In December 2023, NICE approved the combination of olaparib and abiraterone for mCRPC based on the PROpel trial, which demonstrated a significant improvement in radiographic progression-free survival. However, real-world data on the effectiveness and tolerability of this combination are lacking. To our knowledge, this is the first UK-based real-world study evaluating this regimen in contemporary patients.

**Methods:**

We conducted a retrospective chart review of patients with mCRPC treated with abiraterone and Olaparib (Abi-Ola) between December 2023 and June 2025 at 2 UK centers: Portsmouth Hospitals University NHS Trust and Newcastle Hospitals NHS Trust. Demographics, disease characteristics, prior treatments, treatment tolerability, and adverse events were analyzed.

**Results:**

A total of 36 patients were included. The median age was 69 years (52-84). At baseline, 25 patients (69.4%) had ECOG performance status (PS) 0, and 11 (30.5%) had PS 1. The median PSA at diagnosis was 240 ng/mL. Prior treatments, disease setting at mCRPC diagnosis, Abi-Ola treatment discontinuation, dose reduction and toxicity are summarized in the Table. Twenty-eight patients (77.8%) had previous docetaxel. Before initiating therapy, only 2 patients had G1 anemia.

With a median follow-up of 11.1 months (95% CI, 9.9-15.4), 3 patients (8.3%) had died, and 9 (25.0%) discontinued Ola-Abi due to disease progression. Treatment discontinuation due to toxicity occurred in 8 patients (22.2%), with 1 patient stopping both agents and 7 patients discontinuing Ola alone. Dose reductions were required in 12 patients (33.3%): 11 for Ola and 1 for both agents. Anemia was observed in 14 patients (38.8%), with 10 experiencing Grade (G) 1, 2 G2, and 2 G3 severity. The other most common side effects observed were fatigue in 20 patients (55.5%; 13 G1, 7 G2), and nausea in 9 patients (25.0%; all G1).

**Conclusions:**

This real-world analysis suggests that the combination of Abi-Ola is feasible and generally well tolerated in the mCRPC setting, with manageable toxicity profiles similar to those reported in the PROpel trial. Further follow-up and comparison with clinical trial outcomes are warranted to validate efficacy and long-term safety in broader clinical practice and in patients’ subgroups.

**Table:** 

**Category**	**Description**	***N* (of 36)**	**%**	**Notes**
** Previous treatments **	Prostatectomy	6	16.7%	
	Radical radiotherapy to the prostate	13	36.1%	
	Docetaxel chemotherapy	28	77.8%	
	Consolidation prostate radiotherapy	8	22.2%	
** Previous disease setting **	De novo high-volume	12	33.3%	
	De novo low-volume	11	30.5%	
	Metachronous low-volume	9	25.0%	
	Metachronous high-volume	1	2.7%	
	Locally advanced disease	3	8.3%	
** Treatment discontinuation **	Total discontinuations	17	47.2%	
	Disease progression	9	25.0%	Reason for discontinuation
	Toxicity	8	22.2%	Reason for discontinuation
** Dose reductions **	Patients with dose reductions	12	33.3%	11 Ola, 1 both agents
** Common adverse events **	Fatigue	20	55.5%	13 G1, 7 G2
	Anemia	14	38.8%	10 G1, 2 G2, 2 G3
	Nausea	9	25.0%	All G1
	Vomiting	1	2.7%	G1
	Diarrhea	3	8.3%	All G1
	Thrombocytopenia	2	5.5%	1 G1, 1 G3
	Liver function abnormalities	5	13.8%	3 G1, 1 G2, 1 G3
	Acute kidney injury	4	11.1%	All G1
	Cardiac adverse events	6	16.6%	5 G1 and 1 G2
	New-onset diabetes	2	5.5%	-

